# {4-Hy­droxy-*N*′-[(2*E*,3*Z*)-4-oxido-4-phenyl­but-3-en-2-yl­idene]benzo­hydra­zidato}diphenyl­tin(IV) methanol monosolvate

**DOI:** 10.1107/S1600536811023415

**Published:** 2011-06-22

**Authors:** Md. Abu Affan, Norrihan B. Sam, Fasihuddin B. Ahmad, Fraser White, Edward R. T. Tiekink

**Affiliations:** a Faculty of Resource Science and Technology, Universiti Malaysia Sarawak, 94300 Kota Samarahan, Sarawak, Malaysia; b Agilent Technologies UK Ltd, 10 Mead Road, Oxford Industrial Park, Oxford OX5 1QU, England; c Department of Chemistry, University of Malaya, 50603 Kuala Lumpur, Malaysia

## Abstract

Two independent diphenyl­tin mol­ecules and two independent methanol mol­ecules comprise the asymmetric unit of the title compound, [Sn(C_6_H_5_)_2_(C_17_H_14_N_2_O_3_)]·CH_3_OH. The Sn atom in each is five-coordinated by a tridentate ligand and the *ipso*-C atoms of the Sn-bound benzene substituents. The resulting C_2_N_2_O donor set defines a coordination geometry that is inter­mediate between trigonal-bipyramidal (TP) and square-pyramidal (SP), with one mol­ecule slightly tending towards TP and the other slightly towards SP. The mol­ecules differ in terms of the relative orientations of the terminal benzene rings [dihedral angles = 45.71 (18) and 53.98 (17)°] and of the Sn-bound benzene substituents [dihedral angles = 59.5 (2) and 45.77 (18)°, respectively]. The most prominent feature of the crystal packing is the formation of four-mol­ecule aggregates *via* O—H⋯O and O—H⋯N hydrogen bonds, in which the hy­droxy group is connected to a methanol mol­ecule which, in turn, is linked to a non-coordinating N atom. Weak C—H⋯π inter­actions also occur.

## Related literature

For background to the biological inter­est in related compounds, see: Affan *et al.* (2010 ▶). For related structures, see: Affan *et al.* (2009 ▶, 2011 ▶). For additional structural analysis, see: Addison *et al.* (1984 ▶).
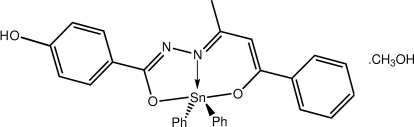


## Experimental

### 

#### Crystal data

[Sn(C_6_H_5_)_2_(C_17_H_14_N_2_O_3_)]·CH_4_O
*M* *_r_* = 599.23
Monoclinic, 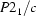

*a* = 18.6824 (2) Å
*b* = 28.7280 (4) Å
*c* = 10.3369 (1) Å
β = 99.856 (1)°
*V* = 5466.02 (11) Å^3^
*Z* = 8
Cu *K*α radiation
μ = 7.74 mm^−1^
*T* = 150 K
0.37 × 0.29 × 0.17 mm

#### Data collection

Agilent SuperNova Dual diffractometer with an Atlas detector
Absorption correction: analytical (*CrysAlis PRO*; Agilent, 2011 ▶) *T*_min_ = 0.231, *T*_max_ = 0.611
17500 measured reflections
9175 independent reflections
8138 reflections with *I* > 2σ(*I*)
*R*_int_ = 0.036

#### Refinement

*R*[*F* ^2^ > 2σ(*F* ^2^)] = 0.032
*wR*(*F* ^2^) = 0.079
*S* = 1.00
9175 reflections
675 parameters
H-atom parameters constrained
Δρ_max_ = 0.46 e Å^−3^
Δρ_min_ = −0.58 e Å^−3^

### 

Data collection: *CrysAlis PRO* (Agilent, 2011 ▶); cell refinement: *CrysAlis PRO*; data reduction: *CrysAlis PRO*; program(s) used to solve structure: *SHELXS97* (Sheldrick, 2008 ▶); program(s) used to refine structure: *SHELXL97* (Sheldrick, 2008 ▶); molecular graphics: *ORTEP-3* (Farrugia, 1997 ▶), *DIAMOND* (Brandenburg, 2006 ▶) and Qmol (Gans & Shalloway, 2001 ▶); software used to prepare material for publication: *PLATON* (Spek, 2009 ▶) and *publCIF* (Westrip, 2010 ▶).

## Supplementary Material

Crystal structure: contains datablock(s) global, I. DOI: 10.1107/S1600536811023415/hb5914sup1.cif

Structure factors: contains datablock(s) I. DOI: 10.1107/S1600536811023415/hb5914Isup2.hkl

Additional supplementary materials:  crystallographic information; 3D view; checkCIF report

## Figures and Tables

**Table 1:** Selected bond lengths (Å)

Sn1—O1	2.124 (2)
Sn1—O3	2.102 (2)
Sn1—N2	2.133 (2)
Sn1—C18	2.118 (4)
Sn1—C24	2.117 (3)
Sn2—O4	2.123 (2)
Sn2—O6	2.094 (2)
Sn2—N4	2.141 (3)
Sn2—C47	2.116 (3)
Sn2—C53	2.124 (3)

**Table 2:** Hydrogen-bond geometry (Å, °) *Cg*1, *Cg*2 and *Cg*3 are the centroids of the C31–C36, C18–C23 and C12–C17 rings, respectively.

*D*—H⋯*A*	*D*—H	H⋯*A*	*D*⋯*A*	*D*—H⋯*A*
O2—H2o⋯O8^i^	0.84	1.81	2.650 (4)	175
O5—H5o⋯O7^ii^	0.84	1.85	2.681 (4)	170
O7—H7o⋯N3^iii^	0.84	2.02	2.830 (4)	163
O8—H8o⋯N1^iv^	0.84	1.98	2.821 (4)	175
C50—H50⋯*Cg*1^v^	0.95	2.91	3.440 (5)	116
C57—H57⋯*Cg*2	0.95	2.84	3.664 (4)	145
C60—H60a⋯*Cg*3^vi^	0.98	2.98	3.886 (6)	155

Symmetry codes: (i) 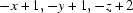; (ii) 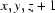; (iii) 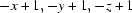; (iv) 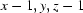; (v) 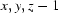; (vi) 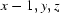.

**Fig. 1.**
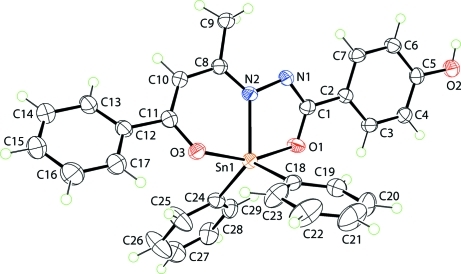
The molecular structure of the first independent molecule of (I) showing displacement ellipsoids at the 50% probability level.

**Fig. 2.**
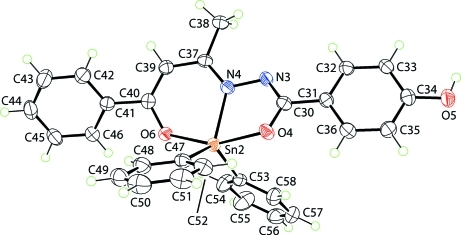
The molecular structure of the second independent molecule of (I) showing displacement ellipsoids at the 50% probability level.

**Fig. 3.**
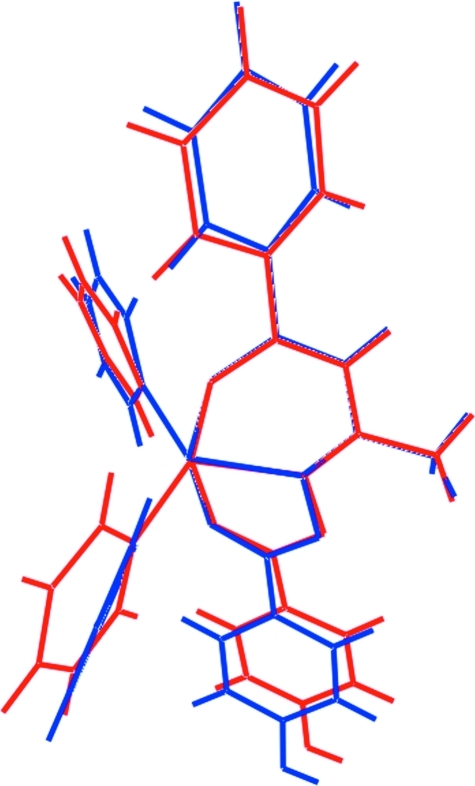
Overlay diagram of the two independent Sn-containing molecules comprising the asymmetric unit of (I). The first independent molecule (with the Sn1 atom) is shown in red.

**Fig. 4.**
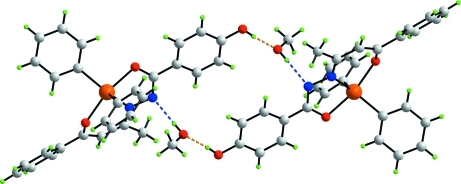
A view of the supramolecular four molecule aggregate of Sn1-containing and methanol molecules in (I). The O—H···O and O—H···N hydrogen bonds are shown as orange and blue dashed lines, respectively.

**Fig. 5.**
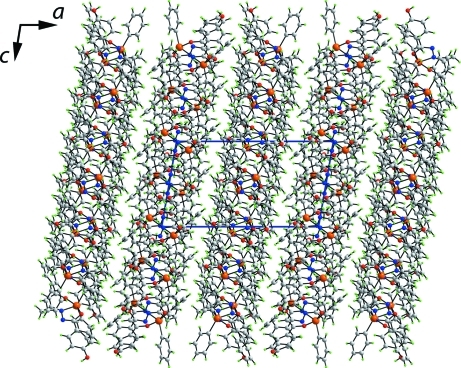
A view in projection down the b axis of the crystal packing in (I) which comprises alternating layers of Sn1 and Sn2 molecules.

## supplementary crystallographic information

### Comment

Motivated by the biological activity of organotin derivatives of biological interest (Affan *et al.*, 2009), the title compound, (I), was examined in connection with complementary structural studies (Affan *et al.*, 2010). The analysis of (I) complements the structure of the dimethyltin derivative (Affan *et al.*, 2011).

Two independent diphenyltin compounds and two methanol molecules of solvation comprise the asymmetric unit of (I). There are some conformational differences between the first independent molecule, Fig. 1, and that of the second, Fig. 2, as discussed below. The Sn atom in each molecule is five-coordinated by the tridentate ligand and two phenyl groups, Table 1. The resulting C_2_NO_2_ donor set defines a coordination geometry intermediate between square pyramidal and trigonal bipyramidal geometry. This is quantified by the value of τ = 0.55 [Sn1] which compares to the τ values of 0.0 and 1.0 for ideal square pyramidal and trigonal bipyramidal geometries, respectively (Addison *et al.*, 1984). The value for the Sn2 atom, τ = 0.47, indicates a small deviation towards square pyramidal. The τ value for the dimethyl derivative of 0.51 (Affan *et al.*, 2011) is intermediate between those calculated for the molecules in (I).

The five-membered SnCN_2_O chelate ring is buckled with a r.m.s. deviation = 0.182 Å and with maximum deviations of 0.116 (1) and -0.144 (2) Å for the Sn1 and N2 atoms, respectively [the equivalent parameters for the second molecule are r.m.s. = 0.224 Å, max. deviations: 0.142 (1) for Sn2 and -0.176 (3) for N4]. There is also considerable distortion in the SnC_3_NO six-membered chelate with the r.m.s. deviation being 0.185 Å, and with the O3 and Sn1 atoms lying 0.214 (2) and -0.160 (1) Å out of the least-squares plane [the equivalent parameters for the second molecule are r.m.s. = 0.222 Å, max. deviations: 0.255 (2) for O6 and -0.191 (1) for Sn2]. The hydroxybenzene ring is slightly twisted out of the plane from the adjacent five-membered chelate ring as seen in the O1—C1—C2—C3 torsion angle of -4.8 (5) ° [an even greater twist is found for the second molecule with O4—C30—C31—C32 being -170.3 (3) °]. By contrast, significant twists are found between the benzene ring and six-membered chelate ring with the O3—C11—C12—C13 torsion angle being -159.6 (3) ° [again, an even greater twist is found for the second independent molecule with O6—C40—C41—C42 = 150.4 (3) °]. The dihedral angle between the terminal benzene rings is 45.71 (18) ° consistent with twist in the tridentate ligand [the equivalent value for the second molecule is 53.98 (17) °]. The aforementioned differences are highlighted in the overlay diagram shown in Fig. 3. The other notable difference between the two independent molecules is found in the dihedral angle formed between the tin-bound benzene rings, *i.e*. 59.5 (2) ° for the Sn1-molecule and 45.77 (18) ° for the Sn2-molecule.

The crystal structure features four molecule aggregates whereby centrosymmetrically related molecules are bridged by methanol molecules. The connections between the molecules are O—H···O hydrogen bonds formed between the benzene-hydroxy group and the methanol-O, and O—H···N hydrogen bonds formed between the methanol and the non-coordinating nitrogen atom, Table 2. The resultant aggregate is cyclic and is stabilized by a 20-membered {···HO···HOC_5_N}_2_ synthon as illustrated for the Sn1 molecule in Fig. 4. Globally, the crystal structure comprises alternating layers made up of Sn1 and Sn2 molecules that stack along the *a* direction and are connected by C—H···π interactions, Table 2.

### Experimental

Benzoylacetone 4-hydroxybenzhydrazone (0.59 g, 2 mmol) was dissolved in distilled methanol (20 ml) under a nitrogen atmosphere. Potassium hydroxide (0.23 g, 4 mmol) dissolved in methanol (10 ml) was added drop wise to the solution during which the colour of the solution changed from yellow to orange. The resulting mixture was refluxed for 1 h, then treated with diphenyltin dichloride (0.687 g, 2 mmol) in methanol (10 ml), heated under reflux for 4 h and allowed to cool to room temperature. Potassium chloride that formed during the reaction was removed *via* filtration. The filtrate was evaporated to dryness using a rotary evaporator to obtain yellow microcrystals. The microcrystals were filtered off, washed with ethanol and dried *in vacuo* over P_2_O_5_ overnight. Yellow blocks of (I) were obtained by slow evaporation of methanol and diethyl ether (1:3 ratio) solution at room temperature. Yield: 1.05 g, 70%. *M*.pt: 455–456 K. IR (ν_max_, cm^-1^, KBr): 3455 (OH), 1596 (C═ N—N═C), 953 (N—N), 564 (Sn—C), 520 (Sn—O), 448 (Sn—N).

### Refinement

Carbon-bound H-atoms were placed in calculated positions (O—H = 0.84 Å; C—H = 0.95 to 0.98 Å) and were included in the refinement in the riding model approximation with *U*_iso_(H) set to 1.2-*U*_eq_(C) and 1.5-*U*_eq_(O, methyl-C).

### Crystal data

**Table:**

[Sn(C_6_H_5_)_2_(C_17_H_14_N_2_O_3_)]·CH_4_O	*F*(000) = 2432
*M**_r_* = 599.23	*D*_x_ = 1.456 Mg m^−^^3^
Monoclinic, *P*2_1_/*c*	Cu *K*α radiation, λ = 1.5418 Å
Hall symbol: -P 2ybc	Cell parameters from 10187 reflections
*a* = 18.6824 (2) Å	θ = 3.1–74.5°
*b* = 28.7280 (4) Å	µ = 7.74 mm^−^^1^
*c* = 10.3369 (1) Å	*T* = 150 K
β = 99.856 (1)°	Block, yellow
*V* = 5466.02 (11) Å^3^	0.37 × 0.29 × 0.17 mm
*Z* = 8	

### Data collection

**Table:**

Agilent SuperNova Dual Cu at zero Atlas diffractometer	9175 independent reflections
Radiation source: SuperNova (Cu) X-ray Source	8138 reflections with *I* > 2σ(*I*)
mirror	*R*_int_ = 0.036
ω scans	θ_max_ = 65.0°, θ_min_ = 3.1°
Absorption correction: analytical (*CrysAlis PRO*; Agilent, 2011)	*h* = −21→21
*T*_min_ = 0.231, *T*_max_ = 0.611	*k* = −27→33
17500 measured reflections	*l* = −12→11

### Refinement

**Table:**

Refinement on *F*^2^	Primary atom site location: structure-invariant direct methods
Least-squares matrix: full	Secondary atom site location: difference Fourier map
*R*[*F*^2^ > 2σ(*F*^2^)] = 0.032	Hydrogen site location: inferred from neighbouring sites
*wR*(*F*^2^) = 0.079	H-atom parameters constrained
*S* = 1.00	*w* = 1/[σ^2^(*F*_o_^2^) + (0.0362*P*)^2^] where *P* = (*F*_o_^2^ + 2*F*_c_^2^)/3
9175 reflections	(Δ/σ)_max_ = 0.002
675 parameters	Δρ_max_ = 0.46 e Å^−^^3^
0 restraints	Δρ_min_ = −0.58 e Å^−^^3^

### Special details

**Table:**

Experimental. Agilent Technologies (2011) *CrysAlis PRO* Software system, version 1.171.34.49, Agilent Technologies UK Ltd, Oxford, UK
Geometry. All s.u.'s (except the s.u. in the dihedral angle between two l.s. planes) are estimated using the full covariance matrix. The cell s.u.'s are taken into account individually in the estimation of s.u.'s in distances, angles and torsion angles; correlations between s.u.'s in cell parameters are only used when they are defined by crystal symmetry. An approximate (isotropic) treatment of cell s.u.'s is used for estimating s.u.'s involving l.s. planes.
Refinement. Refinement of *F*^2^ against ALL reflections. The weighted *R*-factor *wR* and goodness of fit *S* are based on *F*^2^, conventional *R*-factors *R* are based on *F*, with *F* set to zero for negative *F*^2^. The threshold expression of *F*^2^ > 2σ(*F*^2^) is used only for calculating *R*-factors(gt) *etc*. and is not relevant to the choice of reflections for refinement. *R*-factors based on *F*^2^ are statistically about twice as large as those based on *F*, and *R*- factors based on ALL data will be even larger.

### Fractional atomic coordinates and isotropic or equivalent isotropic displacement parameters (Å^2^)

**Table:**

	*x*	*y*	*z*	*U*_iso_*/*U*_eq_	
Sn1	0.913726 (11)	0.613291 (8)	0.89734 (2)	0.02462 (7)	
O1	0.88488 (11)	0.55161 (8)	0.9894 (2)	0.0312 (5)	
O2	0.84683 (13)	0.42008 (9)	1.4635 (2)	0.0372 (6)	
H2O	0.8817	0.4137	1.5236	0.056*	
O3	0.98171 (11)	0.66440 (8)	0.8377 (2)	0.0296 (5)	
N1	1.00051 (14)	0.55638 (10)	1.1106 (2)	0.0267 (6)	
N2	1.01155 (13)	0.58776 (10)	1.0125 (2)	0.0250 (6)	
C1	0.93374 (17)	0.53974 (12)	1.0898 (3)	0.0265 (7)	
C2	0.91311 (16)	0.50704 (11)	1.1859 (3)	0.0245 (7)	
C3	0.84140 (17)	0.49194 (12)	1.1748 (3)	0.0285 (7)	
H3	0.8064	0.5021	1.1026	0.034*	
C4	0.82033 (18)	0.46251 (13)	1.2667 (3)	0.0307 (8)	
H4	0.7711	0.4528	1.2577	0.037*	
C5	0.87068 (17)	0.44702 (12)	1.3725 (3)	0.0290 (7)	
C6	0.94380 (18)	0.46045 (12)	1.3823 (3)	0.0309 (8)	
H6	0.9791	0.4494	1.4528	0.037*	
C7	0.96428 (17)	0.48969 (12)	1.2893 (3)	0.0300 (8)	
H7	1.0139	0.4982	1.2956	0.036*	
C8	1.07935 (16)	0.59773 (12)	1.0015 (3)	0.0256 (7)	
C9	1.14014 (17)	0.57482 (13)	1.0926 (3)	0.0317 (8)	
H9A	1.1415	0.5867	1.1819	0.048*	
H9B	1.1864	0.5817	1.0639	0.048*	
H9C	1.1323	0.5411	1.0918	0.048*	
C10	1.09663 (17)	0.62921 (12)	0.9064 (3)	0.0273 (7)	
H10	1.1460	0.6297	0.8946	0.033*	
C11	1.05056 (17)	0.65907 (12)	0.8290 (3)	0.0265 (7)	
C12	1.07698 (17)	0.68986 (13)	0.7317 (3)	0.0303 (8)	
C13	1.13904 (19)	0.67964 (14)	0.6808 (3)	0.0374 (9)	
H13	1.1653	0.6519	0.7073	0.045*	
C14	1.1632 (2)	0.70882 (16)	0.5929 (4)	0.0492 (11)	
H14	1.2062	0.7013	0.5599	0.059*	
C15	1.1258 (2)	0.74874 (18)	0.5519 (5)	0.0609 (14)	
H15	1.1432	0.7692	0.4921	0.073*	
C16	1.0627 (2)	0.75882 (17)	0.5988 (5)	0.0606 (13)	
H16	1.0359	0.7861	0.5697	0.073*	
C17	1.0382 (2)	0.72947 (15)	0.6878 (4)	0.0456 (10)	
H17	0.9945	0.7365	0.7189	0.055*	
C18	0.85967 (17)	0.66046 (13)	1.0053 (3)	0.0309 (8)	
C19	0.8137 (2)	0.64476 (16)	1.0884 (4)	0.0427 (9)	
H19	0.8083	0.6122	1.1000	0.051*	
C20	0.7761 (2)	0.67520 (19)	1.1540 (4)	0.0555 (12)	
H20	0.7445	0.6639	1.2097	0.067*	
C21	0.7849 (2)	0.7226 (2)	1.1382 (5)	0.0691 (16)	
H21	0.7601	0.7439	1.1851	0.083*	
C22	0.8298 (2)	0.73929 (17)	1.0541 (6)	0.0684 (15)	
H22	0.8348	0.7718	1.0421	0.082*	
C23	0.8670 (2)	0.70802 (15)	0.9878 (5)	0.0494 (11)	
H23	0.8977	0.7192	0.9302	0.059*	
C24	0.87277 (17)	0.59245 (12)	0.7023 (3)	0.0290 (7)	
C25	0.8810 (3)	0.62217 (15)	0.6003 (4)	0.0578 (13)	
H25	0.9056	0.6510	0.6185	0.069*	
C26	0.8534 (3)	0.60990 (18)	0.4715 (4)	0.0714 (16)	
H26	0.8598	0.6300	0.4014	0.086*	
C27	0.8168 (2)	0.56845 (16)	0.4456 (4)	0.0517 (11)	
H27	0.7965	0.5606	0.3576	0.062*	
C28	0.8093 (2)	0.53846 (16)	0.5448 (4)	0.0470 (10)	
H28	0.7853	0.5095	0.5257	0.056*	
C29	0.83686 (18)	0.55053 (14)	0.6737 (4)	0.0368 (8)	
H29	0.8311	0.5299	0.7430	0.044*	
Sn2	0.544563 (10)	0.625910 (7)	0.42618 (2)	0.02395 (7)	
O4	0.58497 (11)	0.56399 (8)	0.5247 (2)	0.0294 (5)	
O5	0.67111 (12)	0.44090 (9)	1.0327 (2)	0.0362 (6)	
H5O	0.6401	0.4291	1.0730	0.054*	
O6	0.46618 (11)	0.67353 (8)	0.3385 (2)	0.0275 (5)	
N3	0.47311 (14)	0.56128 (10)	0.5916 (3)	0.0266 (6)	
N4	0.45350 (13)	0.59189 (10)	0.4855 (3)	0.0263 (6)	
C30	0.54222 (17)	0.54933 (12)	0.6031 (3)	0.0265 (7)	
C31	0.57345 (17)	0.51907 (11)	0.7128 (3)	0.0263 (7)	
C32	0.53045 (17)	0.49582 (12)	0.7912 (3)	0.0296 (7)	
H32	0.4791	0.4983	0.7706	0.035*	
C33	0.56147 (17)	0.46943 (12)	0.8975 (3)	0.0302 (7)	
H33	0.5316	0.4541	0.9499	0.036*	
C34	0.63680 (18)	0.46532 (12)	0.9277 (3)	0.0289 (7)	
C35	0.67993 (18)	0.48745 (12)	0.8492 (3)	0.0315 (8)	
H35	0.7313	0.4845	0.8690	0.038*	
C36	0.64864 (17)	0.51351 (12)	0.7431 (3)	0.0293 (7)	
H36	0.6787	0.5280	0.6895	0.035*	
C37	0.38430 (16)	0.59648 (12)	0.4367 (3)	0.0254 (7)	
C38	0.32879 (17)	0.56738 (13)	0.4907 (3)	0.0344 (8)	
H38A	0.3467	0.5354	0.5044	0.052*	
H38B	0.2830	0.5674	0.4281	0.052*	
H38C	0.3207	0.5805	0.5745	0.052*	
C39	0.36003 (17)	0.62742 (12)	0.3309 (3)	0.0287 (7)	
H39	0.3113	0.6236	0.2873	0.034*	
C40	0.39911 (16)	0.66212 (12)	0.2850 (3)	0.0256 (7)	
C41	0.36756 (16)	0.69069 (12)	0.1693 (3)	0.0265 (7)	
C42	0.31586 (18)	0.67235 (14)	0.0691 (3)	0.0364 (8)	
H42	0.2991	0.6413	0.0752	0.044*	
C43	0.2888 (2)	0.69927 (16)	−0.0395 (4)	0.0457 (10)	
H43	0.2534	0.6865	−0.1072	0.055*	
C44	0.31245 (19)	0.74418 (15)	−0.0508 (4)	0.0403 (9)	
H44	0.2937	0.7624	−0.1256	0.048*	
C45	0.3634 (2)	0.76235 (15)	0.0472 (4)	0.0416 (9)	
H45	0.3801	0.7934	0.0402	0.050*	
C46	0.39095 (19)	0.73594 (13)	0.1562 (3)	0.0345 (8)	
H46	0.4264	0.7490	0.2232	0.041*	
C47	0.58271 (17)	0.60961 (12)	0.2503 (3)	0.0278 (7)	
C48	0.55966 (19)	0.63494 (14)	0.1365 (3)	0.0363 (8)	
H48	0.5259	0.6597	0.1368	0.044*	
C49	0.5859 (2)	0.62421 (15)	0.0219 (4)	0.0431 (10)	
H49	0.5707	0.6419	−0.0556	0.052*	
C50	0.6342 (2)	0.58782 (16)	0.0211 (4)	0.0460 (10)	
H50	0.6519	0.5804	−0.0573	0.055*	
C51	0.6567 (2)	0.56233 (15)	0.1331 (4)	0.0451 (10)	
H51	0.6897	0.5373	0.1317	0.054*	
C52	0.63146 (19)	0.57301 (13)	0.2479 (4)	0.0356 (8)	
H52	0.6474	0.5554	0.3252	0.043*	
C53	0.59273 (16)	0.66994 (12)	0.5821 (3)	0.0271 (7)	
C54	0.5622 (2)	0.71212 (14)	0.6050 (4)	0.0415 (9)	
H54	0.5227	0.7239	0.5435	0.050*	
C55	0.5886 (2)	0.73760 (16)	0.7175 (4)	0.0529 (11)	
H55	0.5663	0.7663	0.7332	0.064*	
C56	0.6464 (2)	0.72180 (15)	0.8055 (4)	0.0456 (10)	
H56	0.6642	0.7394	0.8821	0.055*	
C57	0.6786 (2)	0.68048 (15)	0.7829 (4)	0.0443 (10)	
H57	0.7192	0.6697	0.8435	0.053*	
C58	0.65233 (19)	0.65424 (14)	0.6718 (4)	0.0394 (9)	
H58	0.6750	0.6256	0.6569	0.047*	
O7	0.58272 (16)	0.40602 (10)	0.1873 (3)	0.0475 (7)	
H7O	0.5726	0.4203	0.2528	0.071*	
C59	0.5821 (3)	0.3582 (2)	0.2101 (6)	0.0833 (17)	
H59A	0.6286	0.3488	0.2628	0.125*	
H59B	0.5749	0.3416	0.1261	0.125*	
H59C	0.5425	0.3506	0.2576	0.125*	
O8	0.04591 (17)	0.60516 (10)	0.3473 (3)	0.0524 (8)	
H8O	0.0352	0.5901	0.2770	0.079*	
C60	0.0200 (4)	0.6484 (2)	0.3296 (6)	0.107 (2)	
H60A	0.0245	0.6641	0.4147	0.160*	
H60B	−0.0313	0.6473	0.2884	0.160*	
H60C	0.0477	0.6655	0.2727	0.160*	

### Atomic displacement parameters (Å^2^)

**Table:**

	*U*^11^	*U*^22^	*U*^33^	*U*^12^	*U*^13^	*U*^23^
Sn1	0.02344 (11)	0.02432 (12)	0.02486 (11)	0.00139 (8)	0.00064 (8)	0.00269 (8)
O1	0.0285 (11)	0.0309 (13)	0.0315 (12)	−0.0008 (10)	−0.0028 (10)	0.0085 (11)
O2	0.0355 (13)	0.0391 (15)	0.0374 (13)	−0.0022 (11)	0.0074 (11)	0.0120 (12)
O3	0.0251 (11)	0.0279 (13)	0.0358 (12)	0.0005 (9)	0.0052 (10)	0.0062 (10)
N1	0.0267 (13)	0.0278 (15)	0.0257 (13)	0.0004 (12)	0.0043 (11)	0.0065 (12)
N2	0.0243 (13)	0.0241 (15)	0.0260 (13)	0.0031 (11)	0.0027 (11)	0.0045 (11)
C1	0.0268 (16)	0.0241 (17)	0.0284 (16)	0.0031 (13)	0.0045 (14)	−0.0011 (14)
C2	0.0261 (16)	0.0223 (17)	0.0248 (16)	0.0013 (13)	0.0033 (13)	−0.0003 (13)
C3	0.0253 (16)	0.0310 (19)	0.0282 (16)	0.0019 (14)	0.0014 (13)	0.0009 (15)
C4	0.0271 (16)	0.035 (2)	0.0303 (17)	−0.0036 (14)	0.0058 (14)	0.0004 (15)
C5	0.0318 (17)	0.0277 (18)	0.0284 (17)	−0.0012 (14)	0.0080 (14)	0.0021 (15)
C6	0.0299 (17)	0.0313 (19)	0.0301 (17)	−0.0007 (14)	0.0014 (14)	0.0045 (15)
C7	0.0233 (16)	0.032 (2)	0.0326 (17)	−0.0033 (14)	−0.0004 (14)	0.0047 (15)
C8	0.0254 (16)	0.0274 (18)	0.0238 (15)	0.0025 (13)	0.0033 (13)	−0.0022 (14)
C9	0.0267 (16)	0.034 (2)	0.0345 (18)	0.0035 (14)	0.0048 (14)	0.0078 (16)
C10	0.0254 (16)	0.0298 (19)	0.0270 (16)	0.0001 (13)	0.0055 (13)	0.0021 (14)
C11	0.0305 (17)	0.0251 (18)	0.0236 (16)	−0.0024 (14)	0.0037 (13)	−0.0037 (14)
C12	0.0305 (17)	0.0316 (19)	0.0273 (16)	−0.0035 (14)	0.0007 (14)	0.0065 (15)
C13	0.0359 (19)	0.042 (2)	0.0328 (18)	−0.0054 (17)	0.0022 (16)	0.0101 (17)
C14	0.039 (2)	0.066 (3)	0.043 (2)	−0.002 (2)	0.0076 (18)	0.018 (2)
C15	0.047 (2)	0.070 (3)	0.065 (3)	−0.006 (2)	0.010 (2)	0.038 (3)
C16	0.054 (3)	0.053 (3)	0.074 (3)	0.007 (2)	0.010 (2)	0.035 (3)
C17	0.041 (2)	0.044 (2)	0.052 (2)	0.0016 (18)	0.0099 (18)	0.017 (2)
C18	0.0238 (15)	0.037 (2)	0.0308 (17)	0.0026 (14)	0.0015 (14)	−0.0012 (15)
C19	0.039 (2)	0.053 (3)	0.035 (2)	0.0079 (18)	0.0040 (17)	0.0037 (19)
C20	0.048 (2)	0.080 (4)	0.044 (2)	0.010 (2)	0.0208 (19)	−0.008 (2)
C21	0.050 (3)	0.085 (4)	0.075 (3)	0.013 (3)	0.015 (2)	−0.044 (3)
C22	0.050 (3)	0.042 (3)	0.114 (4)	0.005 (2)	0.016 (3)	−0.029 (3)
C23	0.038 (2)	0.039 (2)	0.074 (3)	−0.0014 (17)	0.018 (2)	−0.011 (2)
C24	0.0308 (16)	0.0282 (18)	0.0266 (16)	0.0033 (14)	0.0012 (14)	−0.0028 (14)
C25	0.092 (3)	0.038 (2)	0.036 (2)	−0.018 (2)	−0.010 (2)	0.0094 (19)
C26	0.111 (4)	0.061 (3)	0.035 (2)	−0.020 (3)	−0.011 (3)	0.010 (2)
C27	0.058 (3)	0.060 (3)	0.033 (2)	−0.006 (2)	−0.0027 (19)	−0.013 (2)
C28	0.048 (2)	0.050 (3)	0.043 (2)	−0.016 (2)	0.0094 (19)	−0.015 (2)
C29	0.0353 (19)	0.038 (2)	0.0384 (19)	−0.0087 (16)	0.0110 (16)	0.0006 (17)
Sn2	0.02155 (11)	0.02523 (12)	0.02636 (11)	0.00062 (8)	0.00779 (8)	0.00407 (9)
O4	0.0268 (11)	0.0286 (13)	0.0354 (12)	0.0052 (10)	0.0127 (10)	0.0096 (10)
O5	0.0348 (12)	0.0356 (15)	0.0378 (14)	0.0066 (11)	0.0049 (11)	0.0128 (11)
O6	0.0223 (10)	0.0278 (13)	0.0315 (12)	−0.0006 (9)	0.0024 (9)	0.0048 (10)
N3	0.0253 (13)	0.0280 (16)	0.0275 (14)	0.0017 (11)	0.0073 (11)	0.0074 (12)
N4	0.0254 (13)	0.0249 (15)	0.0289 (14)	−0.0005 (11)	0.0059 (11)	0.0034 (12)
C30	0.0280 (16)	0.0235 (17)	0.0292 (16)	−0.0001 (13)	0.0083 (14)	−0.0004 (14)
C31	0.0293 (16)	0.0219 (17)	0.0280 (16)	0.0015 (13)	0.0058 (14)	0.0008 (14)
C32	0.0247 (16)	0.0287 (19)	0.0355 (18)	−0.0004 (14)	0.0057 (14)	0.0052 (15)
C33	0.0302 (17)	0.0280 (19)	0.0329 (17)	−0.0021 (14)	0.0072 (14)	0.0068 (15)
C34	0.0349 (18)	0.0228 (17)	0.0292 (17)	0.0033 (14)	0.0061 (14)	−0.0008 (14)
C35	0.0265 (16)	0.033 (2)	0.0362 (18)	0.0057 (14)	0.0079 (14)	0.0016 (16)
C36	0.0291 (17)	0.0262 (18)	0.0352 (18)	0.0031 (14)	0.0124 (14)	0.0020 (15)
C37	0.0219 (15)	0.0260 (18)	0.0298 (16)	−0.0012 (13)	0.0087 (13)	−0.0013 (14)
C38	0.0264 (17)	0.040 (2)	0.0382 (19)	−0.0060 (15)	0.0086 (15)	0.0058 (17)
C39	0.0239 (16)	0.0326 (19)	0.0296 (17)	−0.0001 (14)	0.0047 (14)	0.0011 (15)
C40	0.0273 (16)	0.0261 (17)	0.0243 (15)	0.0048 (13)	0.0071 (13)	−0.0026 (14)
C41	0.0219 (15)	0.0322 (19)	0.0269 (16)	0.0030 (13)	0.0090 (13)	0.0000 (14)
C42	0.0364 (18)	0.034 (2)	0.0373 (19)	−0.0017 (16)	0.0013 (16)	0.0018 (16)
C43	0.041 (2)	0.060 (3)	0.0327 (19)	−0.0002 (19)	−0.0038 (17)	0.0026 (19)
C44	0.0338 (19)	0.056 (3)	0.0326 (19)	0.0110 (18)	0.0101 (16)	0.0173 (18)
C45	0.042 (2)	0.041 (2)	0.043 (2)	−0.0024 (17)	0.0100 (18)	0.0155 (18)
C46	0.0343 (18)	0.035 (2)	0.0330 (18)	−0.0053 (15)	0.0025 (15)	0.0073 (16)
C47	0.0269 (16)	0.0313 (19)	0.0270 (16)	−0.0050 (14)	0.0096 (14)	−0.0030 (14)
C48	0.0356 (19)	0.040 (2)	0.0351 (19)	−0.0049 (16)	0.0098 (16)	0.0001 (17)
C49	0.049 (2)	0.051 (3)	0.0305 (19)	−0.0133 (19)	0.0100 (17)	−0.0024 (18)
C50	0.052 (2)	0.054 (3)	0.037 (2)	−0.011 (2)	0.0229 (18)	−0.017 (2)
C51	0.044 (2)	0.042 (2)	0.054 (2)	0.0010 (18)	0.0232 (19)	−0.012 (2)
C52	0.0373 (19)	0.032 (2)	0.0389 (19)	−0.0010 (16)	0.0121 (16)	−0.0008 (16)
C53	0.0223 (15)	0.0326 (19)	0.0266 (16)	−0.0048 (13)	0.0055 (13)	0.0018 (14)
C54	0.0348 (19)	0.038 (2)	0.047 (2)	0.0061 (17)	−0.0053 (17)	−0.0040 (18)
C55	0.048 (2)	0.044 (3)	0.063 (3)	0.001 (2)	0.000 (2)	−0.019 (2)
C56	0.046 (2)	0.047 (3)	0.043 (2)	−0.0163 (19)	0.0046 (18)	−0.0058 (19)
C57	0.038 (2)	0.050 (3)	0.039 (2)	−0.0063 (18)	−0.0080 (17)	0.0108 (19)
C58	0.0358 (19)	0.037 (2)	0.043 (2)	0.0045 (16)	−0.0006 (17)	0.0052 (17)
O7	0.0659 (18)	0.0409 (16)	0.0417 (15)	0.0091 (14)	0.0260 (14)	0.0102 (13)
C59	0.096 (4)	0.078 (4)	0.082 (4)	0.019 (3)	0.033 (3)	0.006 (3)
O8	0.0736 (19)	0.0423 (17)	0.0349 (14)	0.0028 (15)	−0.0089 (14)	0.0018 (13)
C60	0.123 (5)	0.087 (5)	0.089 (4)	0.028 (4)	−0.040 (4)	−0.027 (4)

### Geometric parameters (Å, °)

**Table:**

Sn1—O1	2.124 (2)	O4—C30	1.302 (4)
Sn1—O3	2.102 (2)	O5—C34	1.357 (4)
Sn1—N2	2.133 (2)	O5—H5O	0.8400
Sn1—C18	2.118 (4)	O6—C40	1.322 (4)
Sn1—C24	2.117 (3)	N3—C30	1.322 (4)
O1—C1	1.305 (4)	N3—N4	1.404 (4)
O2—C5	1.350 (4)	N4—C37	1.312 (4)
O2—H2O	0.8400	C30—C31	1.468 (4)
O3—C11	1.314 (4)	C31—C36	1.395 (4)
N1—C1	1.319 (4)	C31—C32	1.404 (5)
N1—N2	1.399 (4)	C32—C33	1.378 (4)
N2—C8	1.322 (4)	C32—H32	0.9500
C1—C2	1.466 (5)	C33—C34	1.393 (5)
C2—C3	1.394 (4)	C33—H33	0.9500
C2—C7	1.398 (4)	C34—C35	1.392 (5)
C3—C4	1.379 (5)	C35—C36	1.372 (5)
C3—H3	0.9500	C35—H35	0.9500
C4—C5	1.388 (5)	C36—H36	0.9500
C4—H4	0.9500	C37—C39	1.422 (5)
C5—C6	1.407 (5)	C37—C38	1.511 (4)
C6—C7	1.379 (5)	C38—H38A	0.9800
C6—H6	0.9500	C38—H38B	0.9800
C7—H7	0.9500	C38—H38C	0.9800
C8—C10	1.413 (5)	C39—C40	1.368 (5)
C8—C9	1.498 (4)	C39—H39	0.9500
C9—H9A	0.9800	C40—C41	1.486 (4)
C9—H9B	0.9800	C41—C46	1.385 (5)
C9—H9C	0.9800	C41—C42	1.394 (5)
C10—C11	1.371 (5)	C42—C43	1.385 (5)
C10—H10	0.9500	C42—H42	0.9500
C11—C12	1.486 (5)	C43—C44	1.375 (6)
C12—C17	1.383 (5)	C43—H43	0.9500
C12—C13	1.384 (5)	C44—C45	1.370 (5)
C13—C14	1.368 (5)	C44—H44	0.9500
C13—H13	0.9500	C45—C46	1.382 (5)
C14—C15	1.371 (6)	C45—H45	0.9500
C14—H14	0.9500	C46—H46	0.9500
C15—C16	1.380 (6)	C47—C48	1.388 (5)
C15—H15	0.9500	C47—C52	1.394 (5)
C16—C17	1.382 (6)	C48—C49	1.392 (5)
C16—H16	0.9500	C48—H48	0.9500
C17—H17	0.9500	C49—C50	1.381 (6)
C18—C23	1.388 (5)	C49—H49	0.9500
C18—C19	1.389 (5)	C50—C51	1.374 (6)
C19—C20	1.372 (6)	C50—H50	0.9500
C19—H19	0.9500	C51—C52	1.384 (5)
C20—C21	1.385 (7)	C51—H51	0.9500
C20—H20	0.9500	C52—H52	0.9500
C21—C22	1.392 (7)	C53—C54	1.377 (5)
C21—H21	0.9500	C53—C58	1.396 (5)
C22—C23	1.387 (6)	C54—C55	1.391 (5)
C22—H22	0.9500	C54—H54	0.9500
C23—H23	0.9500	C55—C56	1.365 (6)
C24—C25	1.385 (5)	C55—H55	0.9500
C24—C29	1.385 (5)	C56—C57	1.369 (6)
C25—C26	1.389 (6)	C56—H56	0.9500
C25—H25	0.9500	C57—C58	1.391 (5)
C26—C27	1.377 (6)	C57—H57	0.9500
C26—H26	0.9500	C58—H58	0.9500
C27—C28	1.365 (6)	O7—C59	1.394 (6)
C27—H27	0.9500	O7—H7O	0.8400
C28—C29	1.389 (5)	C59—H59A	0.9800
C28—H28	0.9500	C59—H59B	0.9800
C29—H29	0.9500	C59—H59C	0.9800
Sn2—O4	2.123 (2)	O8—C60	1.334 (6)
Sn2—O6	2.094 (2)	O8—H8O	0.8400
Sn2—N4	2.141 (3)	C60—H60A	0.9800
Sn2—C47	2.116 (3)	C60—H60B	0.9800
Sn2—C53	2.124 (3)	C60—H60C	0.9800
			
O3—Sn1—C24	93.22 (11)	C47—Sn2—N4	123.12 (11)
O3—Sn1—C18	94.26 (12)	C53—Sn2—N4	107.98 (11)
C24—Sn1—C18	123.74 (12)	O4—Sn2—N4	73.44 (9)
O3—Sn1—O1	157.92 (8)	C30—O4—Sn2	111.35 (19)
C24—Sn1—O1	96.48 (11)	C34—O5—H5O	109.5
C18—Sn1—O1	96.73 (12)	C40—O6—Sn2	124.2 (2)
O3—Sn1—N2	84.31 (9)	C30—N3—N4	110.7 (3)
C24—Sn1—N2	124.73 (11)	C37—N4—N3	118.1 (3)
C18—Sn1—N2	111.49 (11)	C37—N4—Sn2	128.5 (2)
O1—Sn1—N2	73.86 (9)	N3—N4—Sn2	113.31 (18)
C1—O1—Sn1	112.2 (2)	O4—C30—N3	123.6 (3)
C5—O2—H2O	109.5	O4—C30—C31	117.9 (3)
C11—O3—Sn1	125.9 (2)	N3—C30—C31	118.5 (3)
C1—N1—N2	111.6 (2)	C36—C31—C32	118.0 (3)
C8—N2—N1	117.7 (2)	C36—C31—C30	119.6 (3)
C8—N2—Sn1	128.3 (2)	C32—C31—C30	122.4 (3)
N1—N2—Sn1	114.07 (18)	C33—C32—C31	121.2 (3)
O1—C1—N1	123.3 (3)	C33—C32—H32	119.4
O1—C1—C2	118.5 (3)	C31—C32—H32	119.4
N1—C1—C2	118.2 (3)	C32—C33—C34	119.7 (3)
C3—C2—C7	118.3 (3)	C32—C33—H33	120.1
C3—C2—C1	120.3 (3)	C34—C33—H33	120.1
C7—C2—C1	121.5 (3)	O5—C34—C35	117.5 (3)
C4—C3—C2	121.2 (3)	O5—C34—C33	122.9 (3)
C4—C3—H3	119.4	C35—C34—C33	119.6 (3)
C2—C3—H3	119.4	C36—C35—C34	120.3 (3)
C3—C4—C5	120.3 (3)	C36—C35—H35	119.8
C3—C4—H4	119.8	C34—C35—H35	119.8
C5—C4—H4	119.8	C35—C36—C31	121.1 (3)
O2—C5—C4	118.2 (3)	C35—C36—H36	119.4
O2—C5—C6	122.7 (3)	C31—C36—H36	119.4
C4—C5—C6	119.2 (3)	N4—C37—C39	121.5 (3)
C7—C6—C5	120.0 (3)	N4—C37—C38	119.7 (3)
C7—C6—H6	120.0	C39—C37—C38	118.8 (3)
C5—C6—H6	120.0	C37—C38—H38A	109.5
C6—C7—C2	121.0 (3)	C37—C38—H38B	109.5
C6—C7—H7	119.5	H38A—C38—H38B	109.5
C2—C7—H7	119.5	C37—C38—H38C	109.5
N2—C8—C10	122.3 (3)	H38A—C38—H38C	109.5
N2—C8—C9	119.0 (3)	H38B—C38—H38C	109.5
C10—C8—C9	118.7 (3)	C40—C39—C37	127.1 (3)
C8—C9—H9A	109.5	C40—C39—H39	116.4
C8—C9—H9B	109.5	C37—C39—H39	116.4
H9A—C9—H9B	109.5	O6—C40—C39	124.0 (3)
C8—C9—H9C	109.5	O6—C40—C41	114.6 (3)
H9A—C9—H9C	109.5	C39—C40—C41	121.3 (3)
H9B—C9—H9C	109.5	C46—C41—C42	118.2 (3)
C11—C10—C8	127.6 (3)	C46—C41—C40	120.6 (3)
C11—C10—H10	116.2	C42—C41—C40	121.2 (3)
C8—C10—H10	116.2	C43—C42—C41	120.1 (4)
O3—C11—C10	123.9 (3)	C43—C42—H42	119.9
O3—C11—C12	114.8 (3)	C41—C42—H42	119.9
C10—C11—C12	121.3 (3)	C44—C43—C42	121.0 (4)
C17—C12—C13	118.4 (3)	C44—C43—H43	119.5
C17—C12—C11	119.8 (3)	C42—C43—H43	119.5
C13—C12—C11	121.9 (3)	C45—C44—C43	119.2 (3)
C14—C13—C12	121.0 (4)	C45—C44—H44	120.4
C14—C13—H13	119.5	C43—C44—H44	120.4
C12—C13—H13	119.5	C44—C45—C46	120.5 (4)
C15—C14—C13	120.6 (4)	C44—C45—H45	119.7
C15—C14—H14	119.7	C46—C45—H45	119.7
C13—C14—H14	119.7	C41—C46—C45	121.0 (3)
C14—C15—C16	119.2 (4)	C41—C46—H46	119.5
C14—C15—H15	120.4	C45—C46—H46	119.5
C16—C15—H15	120.4	C48—C47—C52	119.3 (3)
C15—C16—C17	120.3 (4)	C48—C47—Sn2	120.7 (3)
C15—C16—H16	119.8	C52—C47—Sn2	120.0 (3)
C17—C16—H16	119.8	C47—C48—C49	120.1 (4)
C16—C17—C12	120.4 (4)	C47—C48—H48	119.9
C16—C17—H17	119.8	C49—C48—H48	119.9
C12—C17—H17	119.8	C50—C49—C48	119.9 (4)
C23—C18—C19	119.0 (4)	C50—C49—H49	120.1
C23—C18—Sn1	119.6 (3)	C48—C49—H49	120.1
C19—C18—Sn1	121.3 (3)	C51—C50—C49	120.3 (4)
C20—C19—C18	121.5 (4)	C51—C50—H50	119.8
C20—C19—H19	119.3	C49—C50—H50	119.8
C18—C19—H19	119.3	C50—C51—C52	120.3 (4)
C19—C20—C21	119.2 (4)	C50—C51—H51	119.9
C19—C20—H20	120.4	C52—C51—H51	119.9
C21—C20—H20	120.4	C51—C52—C47	120.1 (4)
C20—C21—C22	120.6 (4)	C51—C52—H52	119.9
C20—C21—H21	119.7	C47—C52—H52	119.9
C22—C21—H21	119.7	C54—C53—C58	118.5 (3)
C23—C22—C21	119.5 (5)	C54—C53—Sn2	121.4 (2)
C23—C22—H22	120.3	C58—C53—Sn2	119.8 (3)
C21—C22—H22	120.3	C53—C54—C55	120.5 (3)
C22—C23—C18	120.3 (4)	C53—C54—H54	119.7
C22—C23—H23	119.9	C55—C54—H54	119.7
C18—C23—H23	119.9	C56—C55—C54	120.6 (4)
C25—C24—C29	119.1 (3)	C56—C55—H55	119.7
C25—C24—Sn1	118.8 (3)	C54—C55—H55	119.7
C29—C24—Sn1	122.1 (3)	C55—C56—C57	119.7 (4)
C24—C25—C26	120.1 (4)	C55—C56—H56	120.2
C24—C25—H25	119.9	C57—C56—H56	120.2
C26—C25—H25	119.9	C56—C57—C58	120.5 (3)
C27—C26—C25	119.8 (4)	C56—C57—H57	119.7
C27—C26—H26	120.1	C58—C57—H57	119.7
C25—C26—H26	120.1	C57—C58—C53	120.1 (4)
C28—C27—C26	120.8 (4)	C57—C58—H58	119.9
C28—C27—H27	119.6	C53—C58—H58	119.9
C26—C27—H27	119.6	C59—O7—H7O	109.5
C27—C28—C29	119.7 (4)	O7—C59—H59A	109.5
C27—C28—H28	120.2	O7—C59—H59B	109.5
C29—C28—H28	120.2	H59A—C59—H59B	109.5
C24—C29—C28	120.5 (4)	O7—C59—H59C	109.5
C24—C29—H29	119.7	H59A—C59—H59C	109.5
C28—C29—H29	119.7	H59B—C59—H59C	109.5
O6—Sn2—C47	94.94 (11)	C60—O8—H8O	109.5
O6—Sn2—C53	96.51 (11)	O8—C60—H60A	109.5
C47—Sn2—C53	128.51 (12)	O8—C60—H60B	109.5
O6—Sn2—O4	156.93 (8)	H60A—C60—H60B	109.5
C47—Sn2—O4	94.72 (11)	O8—C60—H60C	109.5
C53—Sn2—O4	93.76 (11)	H60A—C60—H60C	109.5
O6—Sn2—N4	83.75 (9)	H60B—C60—H60C	109.5
			
O3—Sn1—O1—C1	27.1 (4)	O6—Sn2—O4—C30	−31.2 (4)
C24—Sn1—O1—C1	142.6 (2)	C47—Sn2—O4—C30	−145.7 (2)
C18—Sn1—O1—C1	−92.2 (2)	C53—Sn2—O4—C30	85.2 (2)
N2—Sn1—O1—C1	18.2 (2)	N4—Sn2—O4—C30	−22.5 (2)
C24—Sn1—O3—C11	−94.8 (3)	C47—Sn2—O6—C40	88.0 (2)
C18—Sn1—O3—C11	141.0 (2)	C53—Sn2—O6—C40	−142.3 (2)
O1—Sn1—O3—C11	21.3 (4)	O4—Sn2—O6—C40	−26.4 (4)
N2—Sn1—O3—C11	29.8 (2)	N4—Sn2—O6—C40	−34.8 (2)
C1—N1—N2—C8	−162.8 (3)	C30—N3—N4—C37	160.5 (3)
C1—N1—N2—Sn1	16.8 (3)	C30—N3—N4—Sn2	−20.0 (3)
O3—Sn1—N2—C8	−16.0 (3)	O6—Sn2—N4—C37	19.1 (3)
C24—Sn1—N2—C8	74.0 (3)	C47—Sn2—N4—C37	−72.7 (3)
C18—Sn1—N2—C8	−108.3 (3)	C53—Sn2—N4—C37	113.9 (3)
O1—Sn1—N2—C8	160.7 (3)	O4—Sn2—N4—C37	−157.5 (3)
O3—Sn1—N2—N1	164.4 (2)	O6—Sn2—N4—N3	−160.4 (2)
C24—Sn1—N2—N1	−105.6 (2)	C47—Sn2—N4—N3	107.8 (2)
C18—Sn1—N2—N1	72.0 (2)	C53—Sn2—N4—N3	−65.5 (2)
O1—Sn1—N2—N1	−18.9 (2)	O4—Sn2—N4—N3	23.0 (2)
Sn1—O1—C1—N1	−16.5 (4)	Sn2—O4—C30—N3	20.9 (4)
Sn1—O1—C1—C2	162.1 (2)	Sn2—O4—C30—C31	−157.0 (2)
N2—N1—C1—O1	−0.1 (5)	N4—N3—C30—O4	−0.7 (5)
N2—N1—C1—C2	−178.7 (3)	N4—N3—C30—C31	177.2 (3)
O1—C1—C2—C3	−4.8 (5)	O4—C30—C31—C36	10.9 (5)
N1—C1—C2—C3	173.9 (3)	N3—C30—C31—C36	−167.1 (3)
O1—C1—C2—C7	174.3 (3)	O4—C30—C31—C32	−170.3 (3)
N1—C1—C2—C7	−7.0 (5)	N3—C30—C31—C32	11.6 (5)
C7—C2—C3—C4	3.3 (5)	C36—C31—C32—C33	2.0 (5)
C1—C2—C3—C4	−177.7 (3)	C30—C31—C32—C33	−176.8 (3)
C2—C3—C4—C5	−0.4 (5)	C31—C32—C33—C34	−0.5 (5)
C3—C4—C5—O2	176.8 (3)	C32—C33—C34—O5	178.3 (3)
C3—C4—C5—C6	−2.1 (5)	C32—C33—C34—C35	−0.8 (5)
O2—C5—C6—C7	−177.1 (3)	O5—C34—C35—C36	−178.6 (3)
C4—C5—C6—C7	1.8 (5)	C33—C34—C35—C36	0.5 (5)
C5—C6—C7—C2	1.1 (5)	C34—C35—C36—C31	1.0 (5)
C3—C2—C7—C6	−3.6 (5)	C32—C31—C36—C35	−2.2 (5)
C1—C2—C7—C6	177.3 (3)	C30—C31—C36—C35	176.6 (3)
N1—N2—C8—C10	−179.5 (3)	N3—N4—C37—C39	178.8 (3)
Sn1—N2—C8—C10	0.9 (5)	Sn2—N4—C37—C39	−0.6 (5)
N1—N2—C8—C9	0.6 (4)	N3—N4—C37—C38	−3.0 (5)
Sn1—N2—C8—C9	−179.0 (2)	Sn2—N4—C37—C38	177.6 (2)
N2—C8—C10—C11	11.6 (6)	N4—C37—C39—C40	−14.5 (6)
C9—C8—C10—C11	−168.6 (3)	C38—C37—C39—C40	167.2 (3)
Sn1—O3—C11—C10	−29.6 (4)	Sn2—O6—C40—C39	34.4 (4)
Sn1—O3—C11—C12	153.2 (2)	Sn2—O6—C40—C41	−146.4 (2)
C8—C10—C11—O3	3.7 (6)	C37—C39—C40—O6	−3.5 (6)
C8—C10—C11—C12	−179.3 (3)	C37—C39—C40—C41	177.4 (3)
O3—C11—C12—C17	18.9 (5)	O6—C40—C41—C46	−27.2 (4)
C10—C11—C12—C17	−158.4 (3)	C39—C40—C41—C46	152.0 (3)
O3—C11—C12—C13	−159.6 (3)	O6—C40—C41—C42	150.4 (3)
C10—C11—C12—C13	23.0 (5)	C39—C40—C41—C42	−30.4 (5)
C17—C12—C13—C14	2.5 (6)	C46—C41—C42—C43	−0.5 (5)
C11—C12—C13—C14	−178.9 (3)	C40—C41—C42—C43	−178.2 (3)
C12—C13—C14—C15	−0.7 (6)	C41—C42—C43—C44	0.3 (6)
C13—C14—C15—C16	−1.1 (7)	C42—C43—C44—C45	−0.1 (6)
C14—C15—C16—C17	1.2 (8)	C43—C44—C45—C46	0.0 (6)
C15—C16—C17—C12	0.5 (7)	C42—C41—C46—C45	0.5 (5)
C13—C12—C17—C16	−2.3 (6)	C40—C41—C46—C45	178.1 (3)
C11—C12—C17—C16	179.0 (4)	C44—C45—C46—C41	−0.2 (6)
O3—Sn1—C18—C23	14.1 (3)	O6—Sn2—C47—C48	8.8 (3)
C24—Sn1—C18—C23	−82.7 (3)	C53—Sn2—C47—C48	−93.6 (3)
O1—Sn1—C18—C23	174.9 (3)	O4—Sn2—C47—C48	167.8 (3)
N2—Sn1—C18—C23	99.6 (3)	N4—Sn2—C47—C48	94.5 (3)
O3—Sn1—C18—C19	−169.4 (3)	O6—Sn2—C47—C52	−171.1 (3)
C24—Sn1—C18—C19	93.9 (3)	C53—Sn2—C47—C52	86.5 (3)
O1—Sn1—C18—C19	−8.5 (3)	O4—Sn2—C47—C52	−12.1 (3)
N2—Sn1—C18—C19	−83.8 (3)	N4—Sn2—C47—C52	−85.4 (3)
C23—C18—C19—C20	−0.5 (6)	C52—C47—C48—C49	−0.9 (5)
Sn1—C18—C19—C20	−177.1 (3)	Sn2—C47—C48—C49	179.2 (3)
C18—C19—C20—C21	−0.8 (6)	C47—C48—C49—C50	1.0 (6)
C19—C20—C21—C22	1.7 (7)	C48—C49—C50—C51	−0.4 (6)
C20—C21—C22—C23	−1.3 (7)	C49—C50—C51—C52	−0.3 (6)
C21—C22—C23—C18	0.0 (7)	C50—C51—C52—C47	0.3 (6)
C19—C18—C23—C22	0.9 (6)	C48—C47—C52—C51	0.3 (5)
Sn1—C18—C23—C22	177.5 (3)	Sn2—C47—C52—C51	−179.8 (3)
O3—Sn1—C24—C25	−18.0 (3)	O6—Sn2—C53—C54	8.4 (3)
C18—Sn1—C24—C25	79.3 (4)	C47—Sn2—C53—C54	110.1 (3)
O1—Sn1—C24—C25	−178.1 (3)	O4—Sn2—C53—C54	−150.9 (3)
N2—Sn1—C24—C25	−103.3 (3)	N4—Sn2—C53—C54	−77.1 (3)
O3—Sn1—C24—C29	163.2 (3)	O6—Sn2—C53—C58	−178.1 (3)
C18—Sn1—C24—C29	−99.5 (3)	C47—Sn2—C53—C58	−76.5 (3)
O1—Sn1—C24—C29	3.1 (3)	O4—Sn2—C53—C58	22.6 (3)
N2—Sn1—C24—C29	77.9 (3)	N4—Sn2—C53—C58	96.3 (3)
C29—C24—C25—C26	0.1 (7)	C58—C53—C54—C55	−2.2 (6)
Sn1—C24—C25—C26	−178.7 (4)	Sn2—C53—C54—C55	171.3 (3)
C24—C25—C26—C27	1.0 (8)	C53—C54—C55—C56	1.5 (7)
C25—C26—C27—C28	−2.2 (8)	C54—C55—C56—C57	0.0 (7)
C26—C27—C28—C29	2.1 (7)	C55—C56—C57—C58	−0.8 (6)
C25—C24—C29—C28	−0.2 (6)	C56—C57—C58—C53	0.1 (6)
Sn1—C24—C29—C28	178.5 (3)	C54—C53—C58—C57	1.4 (6)
C27—C28—C29—C24	−0.9 (6)	Sn2—C53—C58—C57	−172.2 (3)

### Hydrogen-bond geometry (Å, °)

**Table:**

Cg1, Cg2 and Cg3 are the centroids of the C31–C36, C18–C23 and C12–C17 rings, respectively.

**Table:**

*D*—H···*A*	*D*—H	H···*A*	*D*···*A*	*D*—H···*A*
O2—H2o···O8^i^	0.84	1.81	2.650 (4)	175
O5—H5o···O7^ii^	0.84	1.85	2.681 (4)	170
O7—H7o···N3^iii^	0.84	2.02	2.830 (4)	163
O8—H8o···N1^iv^	0.84	1.98	2.821 (4)	175
C50—H50···Cg1^v^	0.95	2.91	3.440 (5)	116
C57—H57···Cg2	0.95	2.84	3.664 (4)	145
C60—H60a···Cg3^vi^	0.98	2.98	3.886 (6)	155

Symmetry codes: (i) −*x*+1, −*y*+1, −*z*+2; (ii) *x*, *y*, *z*+1; (iii) −*x*+1, −*y*+1, −*z*+1; (iv) *x*−1, *y*, *z*−1; (v) *x*, *y*, *z*−1;  (vi) *x*−1, *y*, *z*.
